# Bioavailability of Magnesium and Potassium Salts Used as Potential Substitutes for Sodium Chloride in Human Nutrition — A Review

**DOI:** 10.1002/mnfr.70227

**Published:** 2025-09-10

**Authors:** Ronja Merschmann, Carlotta Burgmer, Gunter P. Eckert, Anika E. Wagner

**Affiliations:** ^1^ Institute of Nutritional Science Justus‐Liebig University Giessen Giessen Germany; ^2^ Center for Sustainable Food Systems Justus‐Liebig‐University Giessen Giessen Germany

**Keywords:** bioavailability, magnesium, pharmacokinetic, potassium, salt substitutes

## Abstract

Hypertension represents a major risk factor for cardiovascular diseases. As a diet high in sodium chloride is associated with hypertension, so‐called “blood pressure salts” are attracting increasing scientific interest. These are characterized by a partial replacement of sodium chloride by other salts, mainly potassium and magnesium compounds. The aim of this review is to evaluate the bioavailability of potassium and magnesium salts as dietary supplements and to identify potential sodium chloride substitutes. A literature search was conducted in the PubMed database using the PICO scheme. Randomized controlled trials in healthy adults investigating the bioavailability of defined potassium/magnesium salts were included. Potassium chloride and potassium citrate showed good bioavailability irrespective of the route of administration and dose. Magnesium citrate and magnesium chloride showed good bioavailability while magnesium oxide was poorly bioavailable. This may be partly due to its poor solubility in water. The results indicate that potassium chloride and potassium citrate as well as magnesium citrate and chloride are suitable for the use as salt substitutes and for increasing potassium and magnesium intake in addition to reducing sodium. Due to its poor water solubility and consequently low bioavailability magnesium oxide is less suitable.

## Graphical abstract

A high intake of sodium chloride contributes to hypertension. This review evaluates the bioavailability of potassium and magnesium salts as sodium chloride alternatives. Potassium chloride and citrate, as well as magnesium citrate and chloride, show good absorption. In contrast, magnesium oxide has poor bioavailability due to low solubility, limiting its use in reducing dietary sodium.

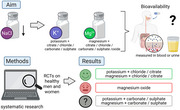

## 1 Introduction

Cardiovascular diseases (CVDs) are the leading cause of global morbidity and mortality. Hypertension is one of the modifiable risk factors for CVDs and has a high prevalence worldwide [1].

Sodium, together with chloride, is one of the components of common table salt and acts as the dominant cation in the extracellular space. Due to its osmotic effect, it is centrally involved in the distribution of total body water. While dietary sodium deficiency and acute sodium excess are rare in the healthy European population, chronic high sodium intake plays an important epidemiological role [2]. The World Health Organization (WHO) recommends a maximum daily intake of 5 g of table salt per day which is exceeded in most European regions, although most studies also assume an underestimation of salt intake [3].

High table salt intake has been associated with various diseases, including hypertension and CVDs [2], while reducing the salt intake to the public recommendations has a therapeutic antihypertensive effect [4]. Several mechanisms have been discussed to explain the relationship between salt and hypertension/CVDs. One concept refers to the salt resistance and sensitivity, which describes an increased peripheral vasoconstriction with a subsequent increase in blood pressure after salt intake in salt‐sensitive individuals compared with non‐salt‐sensitive individuals [5].

Besides a reduction in sodium, an increased potassium intake is considered to have a positive impact on the development of hypertension and CVDs [5]. Potassium is the dominant mineral element within the cell, with the Na^+^/K^+^ ATPase playing a crucial role in maintaining this homeostasis. Like sodium, it is important for fluid distribution inside and outside the cell due to its osmotic effect. Other functions include the regulation of acid‐base balance and the maintenance of electrical activity and muscle contraction, as well as being important in cell metabolism, energy transduction, hormone secretion, and the regulation of protein and glycogen synthesis [6].

The WHO recommends a potassium intake of at least 3.51 g per day for adults, in part to counter the negative effects of high sodium intake on CVDs and heart disease [7]. The European Food Safety Authority (EFSA) estimates the average potassium intake of adults in all countries between 2.46 and 3.99 g daily [6], indicating that the recommendations are not always met.

Hypokalemia and hyperkalemia caused by insufficient food intake are rare [6]. However, dietary intake of potassium has been associated with a number of health outcomes, including reducing hypertension and CVDs. Based on randomized controlled trials, the EFSA Panel expects potassium to have an anti‐hypertensive effect in people with high blood pressure [6].

Magnesium is the second most abundant intracellular cation. It is involved in molecular, biochemical, physiological, and pharmacological processes in the body, such as a cofactor or activator for several enzymes and an essential component of DNA and RNA structures [8]. Based on observations of healthy individuals in the EU, EFSA has established an adequate magnesium intake of 350 mg for adult men and 300 mg for adult women. The EFSA data‐based estimate of magnesium intake in EU countries for adults is in the range of 232–439 mg/day [9]. Hypomagnesemia is usually due to a reduced intake, enhanced excretion, or a combination of these factors and is rare in the general population [8].

Like potassium, an anti‐hypertensive effect has also been discussed for magnesium, although the effect in clinical studies is not consistent. Mechanisms that have been discussed in this context include the use of magnesium as a natural calcium channel blocker through a competition with sodium for binding sites of vascular smooth muscle cells and with an induction of endothelium‐dependent vasodilation. The blood pressure‐lowering effect of magnesium may be improved by a simultaneous increase in potassium intake and a reduction in sodium intake [10].

The use of magnesium and potassium salts could contribute to an increase in potassium and magnesium and a lower sodium intake, which may positively affect health factors, including hypertension and CVDs. A high bioavailability of the salts is of interest to have them systemically available.

In general, bioavailability is often defined as the rate and extent of availability of a drug and its active metabolites in the systemic circulation after absorption. In nutritional science, this definition needs to be adapted because absorption and function depend on factors such as the nutritional and physiological status of the nutrient. Bioavailability in the sense of nutrition refers to the proportion of a substance that can be absorbed and is available for use or storage. Common matrices for the measurement of bioavailability are blood and urine [11].

A high bioavailability is important to ensure a systemic availability of large proportions of ingested magnesium and potassium salts. Therefore, the present review evaluates the bioavailability of various magnesium and potassium salts being suitable as dietary salts. This applies to the potassium salts potassium chloride (KCl), potassium citrate, potassium carbonate and potassium sulfate, which are suitable for the use as food supplements according to Directive 2002/46/EC [12]. The salts magnesium carbonate, magnesium chloride (MgCl), magnesium citrate, magnesium oxide, and magnesium sulfate are included in the Red List for medical purposes and analyzed in this review. They are also used as food supplements for therapeutic purposes and, because of their taste, as alternatives to table salt [13]. The present review investigates how selected potassium and magnesium salts as dietary supplements differ in their bioavailability in healthy adults, which salt compound is a good sodium chloride substitute and looks into the data of side effects of potassium and magnesium salts to provide information on the tolerability.

## 2 Methods

We performed a systematic review following the Preferred Reporting Items for Systematic Reviews and Meta‐Analyses Guidelines. The associated criteria of the PICO scheme are described in Table 1. During the process, the inclusion and exclusion criteria were modified and further confined (see Tables S1 and S2). The final inclusion criteria were as follows: (1) randomized controlled original studies, (2) healthy men and women, (3) normokalemic and normomagnesic values or no indication of deviation (4) selected magnesium or potassium salt compounds that could serve as salt substitute: Magnesium citrate, chloride, carbonate, sulfate, oxide and potassium citrate, chloride, carbonate, sulfate in comparison to an alternative salt substitute (may be other than those mentioned), another formulation, for example, fast versus delayed or another manufacturer (5) urine or blood parameters for evaluation, (6) oral administration of the salt substitutes as a supplement, for example, as tablet or juice. Studies were excluded if they were animal studies or if studies were performed with pregnant or breastfeeding women. In addition, studies with full texts not available in English or German were excluded. The publication and intervention periods were not restricted. Studies including healthy adults were selected unless they explicitly mentioned older adults, children, or adolescents. Normal potassium, magnesium levels, pregnancy, and lactation were assumed absent unless stated otherwise.

**TABLE 1:** Central question of the systematic review defined through the anagram PICO.

Parameter	Inclusion criteria
Population	Healthy men and women between 18 and 65 years
Intervention	Oral administration of a potassium or magnesium salt as a dietary supplement/supplement
Control	Intravenous administration/oral administration of an alternative potassium or magnesium salt
Outcome	Bioavailability measured by serum/plasma parameters or urinary excretion of K^+^ and Mg^2+^

The initial literature search was conducted by CB in the bibliographic database PubMed in February and March 2023, refining the search strategy to the search string below. To update and validate the search, it was independently repeated with inclusion and exclusion criteria directly refined by RM in August 2024 (Tables S1 and S2). The search string, including Boolean operators and truncation (* asterisk) for PubMed was: (potassium AND bioavailability) OR (potassium AND bioequivalen*) OR (“salt substitut*” AND bioavailability) OR (“salt substitut*” AND bioequivalen*) OR (magnesium AND bioavailability) OR (magnesium AND bioequivalen*) OR (dietary AND potassium AND pharmacokineti*) OR (dietary AND magnesium AND pharmacokineti*) OR (dietary magnesium AND bioavailability) OR (dietary potassium AND bioavailability). All references of the included studies were additionally manually screened for further suitable titles.

**FIGURE 1:**
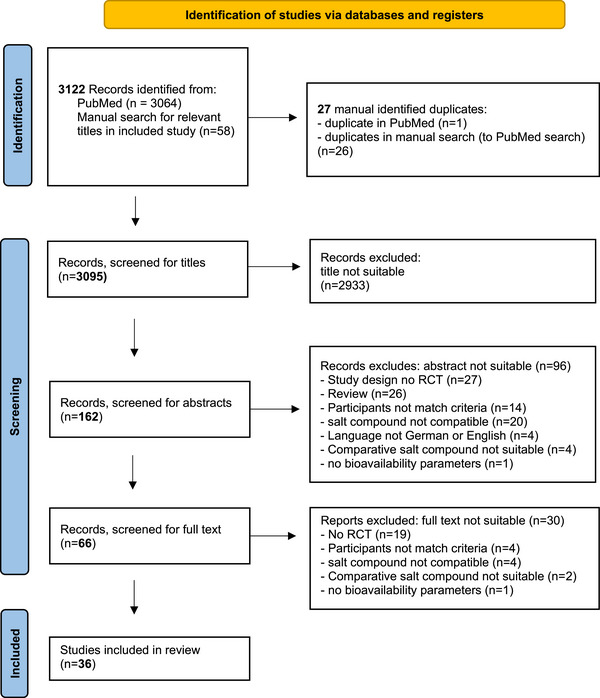
PRISMA (preferred reporting items for systematic reviews and meta‐analyses) flow diagram for study selection. [17]

The search was conducted in PubMed, duplicates were removed, and studies were screened based on titles, abstracts, and full texts. Exclusion reasons were documented. Key data on subjects, salt compounds, doses, study duration, washout phases, matrices, methods, bioavailability, adverse effects, and dietary requirements were extracted. In case of missing age data, alternative measures (e.g., mean) were shown, and study duration was calculated from available data (e.g., treatment days + washout phase in total) if unspecified. Only relevant data are presented. Due to potassium dose variations, absolute excretion values were less comparable, so relative values were calculated (amount excreted/amount ingested * 100) when not provided and marked with a footnote in the table. The risk of bias was assessed using nine criteria from Ding et al. (2015) [14] per Cochrane guidelines. Since all studies involved healthy subjects, the “appropriate cross‐over design” criterion was excluded. Bias was rated high if adverse effects were recorded without blinding, as biomarkers (e.g., urinary K^+^/Mg^2+^ excretion) are less affected by blinding than subjective adverse effects. For parallel‐group studies, the evidence project scale [15] was used (Table S3), assessing factors like Cohort, Control or Comparison Group, Pre/Post Intervention Data, Random Assignment of Participants to the Intervention, Random Selection of Participants for Assessment, Follow‐up Rate of 80% or higher, Comparison Groups Equivalent on Sociodemographic, Comparison Groups Equivalent at Baseline on Outcome measure. The RobVis tool provided a graphical illustration [16]. The search strategy, based on the PRISMA guidelines [17], is shown in Figure 1.

## 3 Results

Twenty‐three studies (63.9%) of the included 36 studies investigated potassium salts and are presented in Table 4. Fourteen studies examined magnesium salts (38.9%) for which the characteristics are shown in Table 5. As König et al. [18] examined both, potassium and magnesium salts, the study is presented in both tables. Single Parts of 16 studies (44.4%) had to be excluded due to missing inclusion criteria and were therefore not presented in the table. Some studies include an in vitro [19, 20, 21, 22, 23, 24, 25] or in vivo [26] study while others investigated additional different matrices such as feces and saliva [27, 28, 29]. Additionally, some sub‐experiments were unsuitable as they were not randomized or lacked a control group [19, 23, 29, 30, 31, 32]. In addition, some studies also tested a group of ill patients [32, 33], or the primary study was included in a review article [34].

All studies provided more or less detailed information on dietary requirements as part of the study, which are summarized in Tables 2 and 3 for K^+^ and Mg^2+^ salts, respectively. Fifteen of the 24 studies on K^+^ salts (62.5%) and 6 of the 14 studies on Mg^2+^ salts (42.9%) also investigated adverse effects, normally as a secondary outcome (Tables 6 and 7). Except for the study by Walker et al. [28] and protocol 2 of Tannen and Cordano [31], which both used a parallel group design, all included studies are crossover studies. Blinding was performed in a total of four studies, from which two studies, both investigating K^+^ salts, were single‐blinded [35, 36] and two studies, both investigating Mg^2+^ salts, were double‐blinded [19, 21]. All other 22 studies (61.1%) did not provide information on blinding. Nearly all studies defined inclusion and exclusion criteria. Thirteen studies (36.1%) only defined “healthy” status as an inclusion criterion [20, 22, 23, 26, 29, 33, 34, 36, 37, 38, 39, 40, 41]. Another 15 studies (41.7%) stated specific diseases as exclusion criteria, for example, gastrointestinal diseases, renal diseases [18, 21, 24, 25, 27, 28, 30, 32, 35, 42, 43, 44, 45, 46, 47]. Overall, the criteria and description of the inclusion and exclusion criteria were inconsistent between the studies. The total number of participants in the studies was between 5 and 64 participants (median: 14, mean ± SD: 17.9 ± 11.5). From the 644 participants included in all studies, 66.8% were male, 24.7% were female, while in 8.5% information on sex was missing. The intervention period (including adaptation/control days and washout periods) ranged from 10 to 60 days (median: 20 days; mean ± SD: 23.6 ± 13), although there were wide variations in the study design. The length of the washout phase varied and ranged between 0 and 8 days (median: 3 days; mean: 3.4 ± 2.4). In some trials, however, the length of the washout period was unclear [23, 25, 28, 30].

**TABLE 2:** Dietary requirements of included studies on potassium salts (per day, if not otherwise specified).

Study	Liquid requirements	Nutrient intake	Other dietary requirements
Arnold et al. (1980) [42]	5200 mL liquid	4920 kcal **100 mEq K** 340 mEq sodium	Forbidden: other food
Bechgaard and Shephard (1981) [38]	Study 1: 1200 mL Study 2: No water load	2000 kcal **<30 mmol K** (for both studies)	Study 1: Individual, identical every day for 9 observation days Study 2: Free choice
Ben‐Ishay and Engelman (1973) [29]	2750 mL distilled water	2200–2400 kcal **50 mEq K**	/
Betlach et al. (1987) [39]	1500 mL liquid	2400 kcal Average of **50 mEq K** and 100 mEq Na	No additional foods or snacks permitted
Caplain et al. (1991) [35]	2500 mL mineral water	2000 kcal **75 mEq K** 150 mEq Na	Forbidden: other food
Dickerson and Bressler (1980) [33]	/	**95 mEq K** 80 mEq Cl	Formula diet
Graffner and Sjögren (1971) [20]	/	/	Avoiding K rich food by written instructions
Harvey et al. (1989) [44]	2500 mL distilled water (on study days)	400 mg Ca 800 mg P 100 mEq Na	Neutral ash
Holzgreve and Stephan (1978) [36]	/	/	Constant food intake and constant intake or avoidance of K rich food like fruit juice, fresh and dried fruits
Koenig et al. (1991) [18]	3 L distilled water	400 mg Ca 800 mg P 100 mEq Na	Breakfast: low in K and citrate
Li et al. (2023) [48]	3000–5000 mL water	2500‐3500 kcal **50‐60 mEq K** 160‐180 mEq Na	/
Lowance et al. (1982) [27]	1500–3200 mL liquid	**Drug‐free days**: 2500 kcal (354 g carbohydrates, 101.4 g fat, 25 g protein) **504 mEq K**, 7.8 mEq Na, 7.9 mEq Cl, 1 mEq/kg body weight supplemental Na **Drug days**: 1256.2 kcal (178.6 g carbohydrates, 55.6 g fat, 22.8 g protein) 2.5 mEq Na, **18 mEq K** 2.5 mEq Cl	/
Melikian et al. (1988) [49]	/	**60 mEq K** 160–180 mEq Na	/
Mittapalli et al. (2017) [22]	3000–5000 mL	**50 to 60 mEq K**	Diet equilibration period from 144 h prior dosing through 48 h prior dosing in each period
Möller et al. (1980) [26]	/	**90 mmol K**	Standardized meals
Otto and Rooth (1973) [23]	/	/	All same diet, no K rich food
Rahkit et al. (1987) [41]	1700 mL	2400 kcal **50 mEq K** 100 mEq NaCl	Each subject same diet
Senel et al. (1991) [24]	2500 mL water	**90 mEq K**	No additional food or snacks permitted
Skouktakis et al. (1979) [51]	/	2200–2400 kcal **70‐80 mEq K** 160–180 mEq NaCl	/
Skoutakis et al. (1985) [50]	/	**60 mEq K** 160–180 mEq NaCl	/
Skoutakis et al. (1984) [34]	/	**65 + 5 mEq K**	/
Tannen and Cordano (1978) [31]	Constant	35 kcal/kg	Formula diet
Toner et al. (1985) [37]	1800 mL	/	**Diet**: individual, identical every day, forbidden: Coffee, tea, alcohol

Abbreviations: Ca, calcium; Cl, chlorine; K, potassium; Mg, magnesium; Na, sodium; p, Phosphorus.

**TABLE 3:** Dietary requirements of included studies on magnesium salts (per day, if not otherwise specified).

Study	Liquid requirements	Nutrient intake	Other dietary requirements
Blancquaert et al. (2019) [19]	Mg free water on test days	/	Prior: no Mg rich food, same meal before each test, no alcohol; test days: standardized meals
Bøhmer et al. (1990) [53]	/	/	Constant food intake on all experimental days, excluding Mg rich items as chocolate, nuts and coffee; no alcohol on test days and 2 days before
Dualé et al. (2018) [21]	80 mL/h on test days**, prior**: 4 days 0.5 L magnesium‐rich water per day	/	Standardized low Mg meals, 3 days prior study no Mg rich food/drinks
Firoz and Graber (2001) [43]	/	/	No Mg rich food like nuts, whole grain bread, dried beans and mineral water during the study
Gegenheimer et al. (1994) [40]	Mg free water	/	/
Kappeler et al. (2017) [45]	Carbonated water with normal Mg content, 2 L per day in standardized regimen	Approximately **300 to 400 mg Mg** per day. (i.e., identical meals)	/
Koenig et al. (1991) [18]	3.25 L distilled water	400 mg Ca 800 mg P 100 mEq Na	Breakfast: low in potassium and citrate
Lindberg et al. (1990a) [32]	2500 mL fluid	400 mg Ca **200 mg Mg** 100 mEq Na	Frozen metabolic diets
Lindberg et al. (1990b) [25]	Evening and night before trial: 300 mL distilled water at 6 p.m. and midnight	**200 mg Mg** 400 mg Ca 800 mg P, 100 mEq Na for 3 days before each load	/
Siener et al. (2010) [52]	Constant intake	2533 kcal (96 g protein, 107 g fat, 290 g carbohydrates) 823g calcium **404 mg Mg** 2500 mg K 4100 mg Sodium	Standardized diet
Walker et al. (2003) [28]	Avoiding/restricting caffeine and alcohol during study	/	Advised to avoid Mg rich food 12 h before and on sampling day, 12 h before and on sampling days
Werner et al. (2019) [46]	/	/	Controlled diet, strictly avoiding Mg rich foods and any beverages including chocolate, dried fruits and nuts, mineral supplements, and Mg rich mineral water
White et al. (1990) [47]	/	/	Standard low Mg diet on the 4 study days, each subject identical diet on all days, no high Mg food such as chocolate, legumes, green vegetables, no caffeine,s dairy products restricted
Wilimzig et al. (1996) [30]	/	**75 mmol** (= 1822.5 mg) **Mg** (prior, for saturation of Mg stores) **4 mmol Mg** (= 97.2 mg) on test days	/

Abbreviations: Ca, calcium; Cl, chlorine; K, potassium; Mg, magnesium; Na, sodium; P, phosphorus.

**TABLE 4:** Characteristics of included studies on potassium salts.

Study / Country	Sample /design	Salt compound	Dose and timing, intervention and trt duration	Sample	Sample collection	Method	Results (Bioavailability)
Arnold et al. (1980) [42] USA	*N* = 12 (12 m) 19–55 y	1) KCl capsule 2) KCl elixir 3) KCl tab.	1) 10 capsules (80 mEq K)^a^ as single dose 2) 3 × 26.6 mEq every 6 h (80 mEq K) 3) 10 tab. (80 mEq K) as single dose **15 days** (1 trt day)^b^	Urine	Control/test days: hourly for 16 h, 16–24 h pooled Remaining days: every 4 h for 16 h	FP	Cumulative net ^c^ 24 h K excretion in mEq (mean ± SD): 1) 40.6 ± 11.4, 2) 43.0 ± 11.4, 3) 50.5 ± 7.6 3) Sign. higher than 1) and 2) Kinetics, max excretion rate mEq/h (mean time): 1) 8.1 (6.8 h), 2) 6.1 (2 h), 3) 10.1 (4 h) 0‐6 h: 3); 6‐12 h: 1); 12‐24 h: 2), each higher than both others to the time BV (%dose ^d^): 1) 50.8%, 2) 53.9%, 3) 63.1%
Bechgaard and Shephard (1981) [38] Denmark	*N* = 13 (5 m, 8 w) 20–51 y	1) Kalinorm tab. (KCl) 2) Slow‐K tab. (KCl) 3) Drug excluded from the report, no further information	**2 studies**: Study 1 with and Study 2 without water‐loading, same supplementation: 1), 2), and 3): 4 tab. single dose (32 mmol) Study 1: **3 weeks** (1 trt day) Study 2: **3 weeks** (1 trt day)	Urine	Control/test days: pooled urine 2 × 12 h	FP	Cumulative net 24 h K excretion (average of 2 studies): ‐ no sign. difference between 1) and 2)^e^ Relative urinary K excretion (average of studies): 1), 2): 50% of the administered dose BV of 1) in relation to 2): Study 1: 109%, Study 2: 117%
Ben‐Ishay and Engelman (1973) [29] USA	*N* = 10 (ns) 23–25 y	1) Slow‐K tablets (KCl) 2) Solution 10% KCl (with coconut flavor)	**Group 1 and 2 (both all 4 trt)**: Trt 1: 5 tab. 1) (40 mEq K) single dose Trt 2: Stepwise increase of 1) over 4 days to 3 tab. (72 mEq K) as max. dose/day Trt 3: 30 mL 2) (40 mEq K) single dose Trt 4: stepwise increase of 2) over 4 days to 3 × 18 mL (72 mEq K) as max. dose/day **15 days** (for each preparation 1 day and 4 days trt separately)	Blood, Urine	Urine: Daily: 24 h collection Control‐days: Basal urine 2 control days/2 test days: Hourly urine collection, additional pooled 4–10 p.m. and 10 p.m.–7 a.m. next day Blood (trt 1 and 3): *t* = 0, 2,7 h	/	Cumulative net 24h K excretion in mEq K (mean ± SEM): Single dose: 1) 11.1 ± 3, 2) 23 ± 4 (sign.)^f^ Urinary BV (% of dose ± SEM): Single dose: 1) 27.4 ± 7.4%, 2) 59.7 ± 1% (sign.) 4 Days: 1) 61 ± 5, 2) 65 ± 3 (not sign.) Urine kinetics: *t* = 1, 2, 3 h: 2) sign. higher; *t* = 4 h and later, not. sign. Blood: results not reported
Betlach et al. (1987) [39] USA	*N* = 25 (25 m) 19–43 y	1) KCl‐tablet 2) KCl‐elixir	1) 4 tab. single dose (80 mEq) 2) 3× every 6 h 26.7 mEq for 1 day (80 mEq) **13 days** (1 trt day)	Urine	All days (except for the last day): total urine Control/Test days: Hourly: until 4 p.m. Pooled: 4 p.m.–12 a.m.	FP	Cumulative net 24h K excretion in mEq, mean (CV): 1) 40.41 (16.51), 2) 42.18 (12.49), not sign. %dose^g^7: 1) 50.5, 2) 52.7 Kinetics, max. rate mEq K/h and time (values for 1st administration of elixir), mean (CV): 1) 4.5 ± 2.64, 2) 3.48 ± 3.42, not sign. 1) 5.5 ± 1.7 h, 2) 2.2 ± 1.7 h, sign.
Caplain et al. (1991) [35] France	*N* = 12 (12 m) Mean: 22 y	1) Micro‐ encapsulated KCl tab. 2) KCl wax matrix tab. 3) KCl solution 4) Placebo (no further specification)	All single dose: 1) 8 × 600 mg (64.8 mmol K) 2) 5 × 1000 mg (67.5 mmol K) 3) 67.5 mL (67.5 mmol K) 4) Placebo **25 days** (1 trt day)	Urine	Test days: *t* = ‐2–0; 0–2; 2–4; 4–6; 6–8; 8–12; 12–24; 24–36; 36–48 h on test days	PM, highly selective liquid membrane electrode)	Cumulative net 24 and 48 h K excretion (mean ± SD): 1) 104.4 ± 19.2 and 179.8 ± 21.2 mmol 2) 114.9 ± 17.3 and 182.5 ± 30.3 mmol 3) 107.6 ± 18.7 and 173.0 ± 19.5 mmol, all not sign. Kinetics: *t *= 0‐4h: 3) increasing, 1) and 2) decreasing *t* = 4–12 h: 1) and 2) increase BV (%dose), based on 48 h cumulative excretion: 1) 78.8%, 2) 79.2%, 3) 69.5% BV (%) compared to 3) (mean ± SEM): 1) 104.6 ± 10.1%, 2) 105.7 ± 20.0%
Dickerson and Bressler (1980) [33] USA	*N* = 18 (18 m) 22–31 y	1) KCl solution 2) KCl tab.	1) 1 tab. dissolved in water (50 mEq K) 2) 5 tab. (50 mEq K) Both as single dose and on 2 separate days **22 days** (1 trt day)	Blood, Urine	Serum: *t* = 0, 1, 3, 5, 8, 12 Urine: *t* = 0–1, 1–3, 3–5, 5–8, 8–12, 12–24 h	Assay (blood), Urine NS	Mean net serum K increase, mEq/L: *t* = 1 h: 1) 5.08, 2) 4.2^h^ (sign.), other times not sign. Peak for 1) after 1 h and for 2) after 3 h Cumulative K net excretion in mEq (mean) 24 h: 1) 159.4, 2) 169.2 (not sign.) %dose^g^: 1) 318.8%^i^ 2) 338.4%^9^ After 12 h: 1) sign. higher
Graffner and Sjögren (1971) [20] Sweden	*N* = 9 (9 m) 22–44 y	1) KCl tab., insoluble matrix 2) KCl tab., insoluble matrix 3) KCl tab., active material 4) KCl tab., active material 5) KCl tab., active material 6) KCl solution in black currant syrup Different manufactures	**Period 1**: 1), 2) and 4) 3×/day for 5 days (9.5 g K/week), control week **Period 2**: 3), 5) and 6) 3×/day for 5 days [10.1 g K/week for 3), 9.5 g/week for 5) and 6)], control week **8 weeks** (2× 4 weeks; 5 trt days, 2 days washout)	Urine	24‐h‐Urine: every day on control/test days	Flame spectroscopy	Mean cumulative net 24 h K excretion (%dose): 1) 95.8, 2) 99.2, 3) 93.2, 4) 86.1, 5) 80.5, 6) 102.2, not sign.
Harvey et al. (1989) [44] USA	*N* = 18 (4 m, 14 w) 24–46 y	1) KCl Wax matrix tab. 2) KCl liquid (dissolved in distilled water) 3) Placebo	1), 2) single dose (60 mEq K) 3): single dose **18 days** (1 trt day)	Urine	Test days: 6–8 a.m. (before administration) Hourly from 8 a.m. to 2 p.m. Pooled: 5–8 p.m., 8 p.m.–8 a.m. (next day)	FP	K excretion (kinetics): 1) vs. 3) sign. higher excretion for all time points after drug administration until 8 p.m. (*t* = 12 h) 2) vs. 3): sign. higher excretion for all time points after drug administration until 8 p.m. (*t* = 12 h) 1) vs. 2): sign. higher between 11 a.m. and 2 p.m., otherwise not sign.
Holzgreve and Stephan (1978) [36] Germany	*N* = 6 (ns) 18–40 y	1) KCL retard tab. 2) KCL retard, enteric coated tab. 3) Placebo tab.	1), 2): 3×/day 1 tab. (40.2 mval KCl) for 1 week 3) 3×/day 1 tab. for 1 week **21 days** (7 trt day)	Urine	3rd‐7nd day each period: 24 h‐urine	/	Urinary K excretion in mval (mean): 1) 93.5, 2) 91.5, 3) 52.2 BV (%dose): 1) 102.5%, 2) 97.5%
Koenig et al. (1991) [18] USA	*N* = 14 (4 m, 10 w) 21–41 y	1) KMgCit tab. 2) KCit tab. 3) KCl tab. 4) Mg_3_Cit_2_ tab.	Single ingestion: 1) 7 tab. (49 mEq K, 24.5 mEq Mg) 2) 10 tab. (50 mEq K) 3) 7 tab. (49 mEq K) 4) 2.5 tab. (25 mEq Mg) **4 weeks** (1 trt day)	Urine	All test days: 6–8 a.m. (before) Hourly from 8 a.m. to 2 p.m. In intervals: from 2–5 p.m., 5–8 p.m., 8 p.m.–8 a.m. (next day)	/	Cumulative 24 h net K/Mg increase (% dose^g^): 1) 23.9 ± 4 mEq K (48.8%) and 14.1 ± 2.7 mg Mg (57.6%) 2) 26 ± 3.3 mEq K (52%) 3) 20.2 ± 2.9 mEq K (41.2%) 4) 16.5 ± 3.8 mg Mg (66%) ‐ no sign. difference (K and Mg)
Li et al. (2023) [48] China	*N* = 64 T1: (24 m,8 w) T2: (20 m, 12 w) 18–45 y	1) KCl sustained‐release tab. 2) KCl sustained release tab. 3) KCl sustained‐release tab. 1) – 3) from different manufactures, 3 as reference	Test 1: difference between 1) and 3) Test 2: difference between 2) and 3) Both test: 1)–3): 6 g KCl single dose **42 days** (1 trt day)	Urine	On baseline/test days: *t* = 0–1, 1–2, 2–4, 4–6, 6–8, 8–12, 12–15, 15–24 h	OES, inductively coupled	Cumulative K in 24 h K excretion (mg, Ratio and 90%‐CI): Test 1: 1) 1996.5, 3) 1996.5 (Ratio 100%, 93.3‐107.2) Test 2: 2) 1432.1, 3) 1450.8 (Ratio 101.3%, 94.6‐108.5) Both Tests: urinary excretion rate similar, bioequivalent
Lowance et al. (1982) [27] USA	*N* = 12 (12 m) 18–25 y	1) KCL slow‐release tab. 2) KCl solution	1) 6 tab. (48 mEq K) single dose 2) 36 ml (48 mEq K) single dose **16 days** (1 trt day)	Urine	Control day and trt day: 6 p.m.—6 a.m., hourly Day after trt: 6–10 a.m., 10 a.m.–2pm., 2–6 p.m., 6–10 p.m., 10 p.m.–6 a.m Other days: 24 h Urine	FP	Mean hourly K excretion (kinetics): First 3 h: 2) sign. greater than 1) After 5 h: 1) sign. higher than 2) T ½ values (time required for 50% of total administered amount of potassium excreted in urine): 2) 30 min. later than 1)
Melikian et al. (1988) [49] USA	*N* = 28 (28 m) 20–40 y	1) KCl suspension 2) KCl capsule 3) KCl solution	1), 2), 3): single dose in the morning (40 mEq) **20 days** (1 trt day)	Urine	Intervention day: *t* = 0–2, 2–4, 4–6, 6–8, 8–12, 12–24 h Control phase: 24 h urine	FP	Cumulative 48 h K mEq excretion (mean ± SD): 1) 117.2 ± 15.6, 2) 114.4 ± 14.03, 3) 121.7 ± 15.8 3) higher than 2), sign. difference to baseline Estimated absorption in 24 h in mEq (%dose): 1) 22.6 (56.5%), 2) 19.1 (47.8%), 3) 5.5 (63.8%) Urine excretion (kinetics): 0–2 h: 3) sign. higher than 1) and 2) 4–8 h: 1) sign. higher than 3), after 8 h not sign.
Mittapalli et al. (2017) [22] USA	*N* = 14 (14 m) ns (adult male subjects)	1) KCl, fast‐release tab. 2) KCl, intermediate release tab. 3) KCl, slow‐release tab.	1)–4): 20 mEq K, single dose in the morning **24 days** (1 trt day)	Urine	Control days: every 1–4 h, pooled after 4 P.m. Trt day: *t* = 0–1, 1–2, 2–4, 4–6, 6–8, 8–12, 12–16, 16–24, 24–48 h	OES, validate dilution method	Cumulative urinary 24 h KCL excretion, mean mEg (%CV)^j^: 1) 46.2 (18), 2) 47.7 (20), 3) 36.7 (25) 1) and 2) sign. higher than 3), otherwise not sign. %dose (net excretion)^k^: 1) 58, 2) 60, 3) 46 Kinetics, max. rate mEq K/h,%CV (mean time, range): 1) 6.9, 23 (3 h, 1.5–7 h); 2) 6.8, 19 (5 h, 3–7 h); 3) 5.2, 33 (5 h, 3–7 h), no information to sign.
Möller et al. (1980) [26] Germany	*N* = 10 (4 m, 6 w) 22–42 y^l^	1) KCl retard 2) KCl retard different manufactures	1) 40 mmol K as single dose (5x) 2) 40 mmol K as single dose (5x) **11 days** (1 trt day)	Urine	Control day and trt day: t = 1, 2, 3, 4, 5, 6, 7, 8, 10, 12 and 24h Other days: 24h urine	AAS with PM	Urinary 24 h K excretion in mmol, mean (%CV): 1) 132.9 (9.5), 2) 137.4 (9.6) Cumulative 24 h K net excretion in mmol (%CV): 1) 38.9 (37.8), 38.5 (35.5), not sign.
Otto and Rooth (1973) [23] Sweden	*N* = 11 (ns) 21–36 y	1) KCl, sustained‐release 2) KCl, sustained‐release 3) KCl, insoluble matrix type 1) and 2) different preparations, manufactured for purpose	1)‐3) 2× day (40 mmol K) for 1 week **4 weeks** (7 trt day)	Urine	Urine: 24 h‐Urine for first 4 of the 7 days per week	AAS	Total and net 24 h K excretion (%dose^g^)in mmol (mean SE): 1) 97.7 ± 3.2; 38.6 ± 3.2 (96.5%) 2) 97.7 ± 2.6; 38.6 ± 3.7 (96.5%) 3) 96.0 ± 3.1; 36 ± 5.6 (90%) ‐ no sign. difference between trt
Rahkit et al. (1987) [41] USA	*N* = 24 (24 m) ns (normal healthy male volunteers)	1) KCl solution 2) KCl, slow‐release tab. (new) 3) Slow‐K tablet (KCl)	1) 30 mL (40 mEq K) single dose 2) 1 g (40 mEq K) single dose 3) 5 tab. 600 mg (40 mEq K) single dose **13 days** (1 trt day)	Urine, Blood	Urine, control day and trt day: 8 a.m.–4 p.m., hourly 8 p.m.–12 a.m., collected Urine, 1–2 days before control day: *t* = 0, 1, 3, 6 h Blood: *t *= 0, 1, 3, 6 h	FM	Mean net Urinary K excretion mEq in 24 h and 48 h and %dose^m^ 1) 27.6 mEq (69%), 35.2 mEq (88%) 2) 23.1 mEq (57%), 34.0 mEq (85%) 3) 28.1 mEq (70%), 37.9 mEq (94%) ‐ no sign. differences between trt Blood: no data provided for serum
Senel et al. (1991) [24] Turkey	*N* = 10 (2 m, 8 w) 20–39 y	1) KCl, 20 HPMC 2) KCl, 20% Lubritab 3) KCl, commercially available 4) KCl, enteric coated All sustained release tab.	Single dose: 1) 5 tab. single dose (40 mEq K) 2) 5 tab. single dose (40 mEq K) 3) 5 tab. single dose (40 mEq K) 4) 6 tab. single dose (46 mEq K) **12 days** (1 trt day)	Urine	Control and trt days: Hourly for 8 h Every two hours until 12 h 12–24 h: pooled	FP, lithium as standard	Cumulative 24 h net K excretion in mEq %dose (mean ± SD): 1) 30.5 ± 12.9 and 76.2 ± 32.1% 2) 37.9 ± 8.8 and 94.8 ± 8.8% 3) 36.3 ± 11.5 and 90.9 ± 28.8% 4) 44.6 ± 6.8 and 96.7 ± 12.9%, all not sign. different Kinetics (net increase in time interval): 0‐4h: 1)–3) higher than 4); 2) higher than 3) 4–6 h: not sign. 6–8 h: 4) higher than 1)–3), 3) higher than 1) 24 h: 4) higher than 1)–3)
Skoutakis et al. (1985) [50] USA	*N* = 28 (ns) 20–29 y	1) Micro‐K capsules (KCl) 2) Slow‐K tab. (KCL) 3) Kaochlor S‐F Solution (KCl) 4) control: no drug	1) 5 × caps. single dose (40 mEq) 2) 5 × tab. single dose (40 mEq) 3) 30 mL solution single dose (40 mEq) **4 weeks** (1 trt day)	Urine	Test days: *t* = 0–1, 1–2, 2–4, 4–6, 6–8, 8–12, 12–24 h Control days and day after trt: 24 h Urine	/	Urinary K excretion (mEq K): No sign. trt differences, all higher than control Total amount of urine potassium recovered in 24h: 1) 738%, 2) 74,3%, 3) 72% K excretion rates (mEq K per time interval): *t *= 1,2,4 h: 3) sign, higher; later: 1) and 2) higher
Skoutakis et al. (1979) [51] USA	*N* = 18 (18 m) 21–42 y	1) KCl slow‐release tab. 2) KCl solution 3) KCl solution	1) 6 tab. single dose (40 mEq K) 2) 15 mL single dose (40 mEq K) 3) 30 mL single dose (80 mEq K) **15 days** (1 trt day)	Urine	Trt days: *t* = 0–2, 2–4, 4–6, 6–8, 8–24 Pre‐ and post‐trt: 24 h‐Urine	Technicon Idee sample identification system	Cumulative 24 h K excretion in mEq (mean ± SE): 1) 107.3 ± 4.83, 2) 99.6 ± 6.81, 3) 122 ± 7.21 3) sign. higher than 2) K excretion kinetics: *t* = 0–2 and 2–4 h: 1) 19 vs. 15.1 %dose, 2) 13.8 vs. 9.8 %dose; 1) for both times sign. higher *t* = 8–24 h 1) 55 %dose, 2) 41%dose (sign.)
Skoutakis et al. (1984) [34] USA	*N* = 25 (25 m) ns (healthy male volunteers)	1) KCl wax matrix tab. 2) KCl wax matrix tab. 3) KCl liquid 4) control: no drug	1) 6 tab. single dose (40 mEq K) 2) 4 tab. single dose (40 mEq K) 3) 15 mL single dose (40 mEq) **20 days** (1 trt day)	Urine, blood	Urine: Trt days: *t *= 0–2, 2–4, 4–6, 6–8, 8–12, 12—24 h Control days and post‐trt day: 24‐h‐sample Blood: *t* = 0, 0.5, 1, 1.5, 2, 3, 4, 6, 8 h	/	Urinary excretion (kinetics), peak excretion: 1), 2): *t* = 4–6 h; 3): *t *= 2–4 h %dose (based on Urine excretion): 1)–3): bioequivalent Serum K (kinetics), peak increase: 1), 2): *t* = 4h; 3: *t* = 1.5 h
Tannen and Cordano (1977) [31]	*N* = 14 (14m)^n^ 18–26 y	**Study 1 (CO)** 1) KCl solution 2) KCl tablets 3) water alone **Study 2: (parallel group)** 1) or 2) (as above)	**Study 1**: 1), 2): single dose (50 mmol K) (N = 14) **Study 2**: 1), 2): 3 times/day for 7 days (75 mmol K) (*N* = 8) **Study 1**: 12 days (1 trt day) **Study 2**: 19 days (7 trt days)	Urine, Blood	**Study 1**: Urine: hourly after administration 9.a.m.–5p.m. Blood: 10 a.m. and 2 p.m. **Study 2**: Daily Urine and frequent venous blood sample, blood labeled with Chromium 51	/	**Study 1**: Urinary K excretion after ingestion, mean excretion rates µmol/min (SE) between 9 a.m. and 5 p.m.: 1) 186 (25.2), 2) 174 (30.5), no sign. difference Urine kinetics: *t* = 1–3 h: 1) sign. higher than 2), *t* = 5 h 2) higher than 1) Plasma K in mM and (SE) 1 h after trt: 1) 4.87 (0.17), 2) 4.24 (0.07), sign. difference, ‐ otherwise, no difference, no difference after 5 h **Study 2**: Urinary K excretion in mmol (SE): 1) 177 (20.2) vs. 2) 173 (12.5), not sign. different Plasma K: 2) and 3) not sign. different
Toner et al. (1985) [37] Great Britain	*N* = 5 (5 m) 23–25 y	1) KCl, sugar‐free syrup 2) KCl, sugar‐coated wax tablets Slow‐K 3) water (placebo)	1) 64 mL (64 mmol) single dose 2) 8 tab. (64 mmol) single dose 3) single dose **4 weeks** (1 trt day)	Blood, Urine	Blood: *t* = 0, 10, 20, 30, 40, 50, 60, 90, 120, 180, 300, 420, 600, 1440 min Urine: 4 × 2 h, 1 × 4 h, 1 × 24 h fraction	FP	Mean plasma K: 2) vs. 3) sign. higher in total and in the first 3h Mean plasma K AUC, mean ± SD (mmol^−1^ min): 1) 161 ± 248, 2) 103 ± 276, no sign. difference Urinary K excretion: 1) sign. higher then 3) until *t* = 4 h Mean Urinary BV (range), %dose: 1) 99.3% (35.0–132.8), 2) 88.3% (29.9–95.5), not sign.

Abbreviations: AAS, atomic absorption spectrometry; BV, bioavailability; Cit, citrate; CV, coefficient of variation; FP, flame photometer; HPMC, hydroxypropylmethylzellulose; K, potassium; KCit, potassium citrate; KCL, potassium chloride; KMgCit, potassium magnesium citrate; Lubritab, hydrogenated vegetable oil; m, men; Mg, magnesium; Mg0, magnesium oxide; MgCit, magnesium citrate; MgCl, magnesium chloride; ns, no specification; OES, optical emission spectroscopy; PM, photometric measurement; SD, standard derivation; SEM, standard error of mean; sign., significant; *t*, time; tab., tablets; trt, treatment; w, women.

^a^ Unless otherwise specified, the total administered dose (of all tab./capsules) is given in parentheses

^b^ Unless otherwise specified, trt days are the number of days for each trt

^c^ Unless otherwise specified, net excretion refers to the difference compared to the baseline values (e.g. control week, placebo)

^d^ Unless otherwise specified, % of administered dose

^e^ Data only provided as mean

^f^ No data provided for 4 days.

^g^ Self‐calculated (cumulative net excretion/administered dose * 100)

^h^ The value 4.2 was read from the graph as information was not provided in the text

^i^ Implausible values self‐calculated from data provided in the text.

^j^ abbreviation CV not explained in the publication, possibly refers to coefficient of variation

^k^ data read from figure

^l^ the range was not specified by the authors and was read from the age figures given for all participants

^m^ There were discrepancies between the text and the abstract for this information; reference was made to the information in the text

^n^ based on the total number of participants, not all completing both sub‐studies

**TABLE 5:** Characteristics of included studies on magnesium salts.

Study/country	Sample/design	Salt compound and administration	Dose and timing, intervention, and trt duration	Sample	Sample collection	Method	Results (Bioavailability)
Blancquaert et al. (2019) [19] Belgium	*N* = 30 (15 m, 15 w) Mean: 24.7 y (m) 23.1 y (w)	1) Mg0 and Mg‐glycerophosphate tablet 2) Mg0 tab.	Single dose: Trt 1: 1 tab. 1) (196 mg Mg) Trt 2: 2 tab. 1) (392 mg Mg) Trt 3: 1 tab. 2) 1 (450 mg Mg) **17 days** (1 trt day)	Blood, Urine	Blood: *t* = –60, 0, 30, 60, 90, 120, 150, 180, 240, 360 min Urine: first 6 h after supplementation in one container and the following 18 h in another container	PM	Urine Mg excretion: no difference at all Max. increase in serum and incremental area under curve: Trt 1 and Trt 2 vs. Trt 3: sign. higher for both Trt 1 vs. Trt 2: no sign. difference for both Max. % net in increase in Mg levels: Trt 1: 6.2%; Trt 2: 8%; Trt 3: 4.6%
Bøhmer et al. (1990) [53] Sweden	*N* = 18 (18 w) 22–40 y	1) Mg‐lactate and MgCit, chewable tab. 2) Mg‐lactate and MgOH_2_, chewable tab. 3) MgOH_2_, Emgesan 4) MgCl solution 5) Placebo tab.	1) 3 tab./day (15 mmol Mg) ^a^ 2) 3 tab./day (15 mmol Mg) 3) 2/day (20.6 mmol Mg) 4) 3/day 10 ml (15 mmol Mg) 5) 3 tab./day, 2 test days All 3 times/day **21 days** (2× 1 trt day) ^b^	Urine	Test days: 24‐h collection in 2l bottles (excluding first morning urine on experimental day and including the urine of the following morning)	AAS	Mg net^c^ excretion (mmol/24 h, mean ± SEM) and %dose^f^ 1) 4.91 ± 0.36 (32.7%) 2) 4.51 ± 0.39 (30.1%) 3) 4.53 ± 0.24 (22%) 4) 4.51 ± 0.32 (30.1%) 5) 3.63 ± 0.26, mean from 2 days ‐ no sign. difference between the trt, all higher than placebo
Dualé et al. (2018) [21] France	*N* = 12 (12 m) 18–50 y	1) MgCl tab. 2) Mg‐Carbonat tab. 3) Mg‐rich mineral water	Single dose: 1) 2 tab. (100 mg Mg) 2) 3 tab. (300 mg Mg) 3) 80 mg/day for 4 days before study for Mg loading **11 days** (1 trt day)	Blood, Urine, Erythrocyte	Test days: Blood: *t* = 0; 0.5; 1; 2; 3; 4; 6; 8; 10 h Urine: *t* = 0–5, 5–10, 10–24 h	AAS	Cumulative 24 h urinary net Mg excretion in mg and %dose^f^ 1) 105.5 (105.5%), 2) 117.7 (39.2%), very similar 1) in relation to 2): 76% (0–5 h), 89% (0–10 h), and 87% (0–24 h) Mg in plasma and erythrocytes: Large intra‐subject variability of 90%–2500% in plasma and erythrocytes
Firoz and Graber (2001) [43] USA	*N* = 16 (8 m, 8 w) 22–55 y	1) MgO caps 2) MgCl tab. 3) Mg‐lactate tab. 4) Mg aspartate	Single dose: 1) 1 cap. 3×/day (21.12 mEq) 2) 2 tab. 2×/day (21.2 mEq) 3) 1 tab. 3×/day (21 mEq) 4) 2 tab. 2×/day (21.64 mEq) **12 days** (1 trt day)	Urine	Test days/Control periods: 24 h urine (excluding first morning urine on experimental day and including the urine of the following morning)	PM	Urine 24 h Mg excretion in mg (mean ± SEM) 1) 90.1 ± 9, 2) 110.9 ± 10, 3) 109.9 ± 11.6 4) 105.3 ± 12.6, sign. to baseline only 2)–4) BV (%dose, based on net excretion): 1) 4%, 2)–4): 9%–11%, sign. difference
Gegenheimer et al. (1994) [40]	*N* = 18 (18 m) 24–40 y	1) Mg0 2) Mg, chewable tab. (Mg carbonate) 3) Mg. granulate (Mg carbonate, Mg0)	1) on 5 days before trt, 37.2 mval/day, for satiation of Mg status 2) 1 capsule as single dose (720 mg Mg) 3) 1012 mg as single dose **10 days** (1 trt day)	Urine	Trt days: *t* = 0–2, 2–4, 4–6, 6–8, 8–10, 10–12, 12–16, 16–24	AAS	Urinary 24 h Mg excretion in mval [mean (%dose)^d^] 2) 18.92 (63.07%), 3) 18.06 (60.3%), not sign. Geometric mean of individual ratio of 24 h‐Mg‐excretion of 2)/3) and 90% CI, data log transformation: 103.91 (99.84%–122.65%), bioequivalent
Kappeler et al. (2017) [45] Germany	*N* = 20 (20 m) 18–45 y	1) MgCit capsule 2) MgCit capsule 3) Mg oxide capsule, Mg biolectra	1) 5×/day (500 mg Mg) for 5 days, for satiation of Mg status before trt 2) 2 capsules as single dose (300 mg Mg) 3) 1 capsule as single dose (300 mg Mg) **12 days** (1 trt day)	Urine, Blood, blood cells	Urine, Trt, and baseline days: *t* = 0–2, 2–4, 4–6, 5–8, 8–10, 10–12, 12–14, 14–16, 16–24 Blood, Trt days: pre‐dose and *t* = 1, 2, 3, 4, 5, 6, 9, and 24 h post‐dose	OES, AAS, PM	24 Urine Mg excretion in mmol (mean and SD), mean difference between trt and 95%‐CI: 2) 7.2 ± 1.48), 3) 6.7 ± 1.43; difference: 0.565 (0.212–0.918) Serum‐Mg: *t *= 2–6, 2) sign. higher than 3), otherwise not sign. Mg in blood cells (erythrocytes monocytes, lymphocytes): Not sign. for all time points and blood cells
Koenig et al. (1991) [18] USA	*N* = 14 (4 m, 10 w) 21–41 y	1) KMgCit tab. 2) KCit tab. 3) KCl tab. 4) Mg_3_Cit_2_ tab.	Single ingestion: 1) 7 tab. (49 mEq K, 24.5 mEq Mg) 2) 10 tab. (50 mEq K) 3) 7 tab. (49 mEq K) 4) 2.5 tab. (25 mEq Mg) **4 weeks** (1 trt day)	Urine	All test days: 6–8 a.m. (before) Hourly from 8 a.m. to 2 p.m. In intervals from 2–5 p.m., 5–8 p.m., 8 p.m.–8 a.m. (next day)	/	Cumulative 24 h net K/Mg increase (% dose^f^): 1) 23.9 ± 4 mEq K (48.8%) and 14.1 ± 2.7 mg Mg (57.6%) 2) 26 ± 3.3 mEq K (52%) 3) 20.2 ± 2.9 mEq K (41.2%) 4) 16.5 ± 3.8 mg Mg (66%), no sign. difference (K and Mg)
Lindberg et al. (1990a) [32] USA	*N* = 7^e^ (2 m, 5 w) 28–41 y	1) Mg_3_Cit_2_ tab. 2) Mg0 tab.	Study 1 (empty stomach, all 7 days): 1), 2) 4×/day (40 mEq) **46 days** (7 trt days)	Urine	Last 2 days of each phase: 24‐h‐Urine	AAS	Urinary K excretion (mg/day): 1) 152 ± 36, 2) 155 ± 33 (not sign.)
Lindberg et al. (1990b) [25] USA	*N* = 17 (6 m, 11 w) 22–40 y	1) MgCit tablet 2) Mg0 tablet 3) Distilled water (control)	1) and 2): each 25 mmol (121.6 mg of elemental magnesium) single dose **12 days** (1 trt day)	Blood, Urine	Urine: 6–8 a.m., 8–10 a.m., 10–12 a.m. Blood: ns	AAS	Net Urinary Mg, pooled 8–12 a.m. (mean and SEM): 1) 0.022 ± 0.004, 2) 0.006 ± 0.004 mg/mg creatinine (sign.) Serum Mg, pooled 8–12 a.m. (mEq/L): 1) 0.126 ± 0.270, 2) 0.028 ± 0.014 mEq/L (sign.)
Siener et al. (2011) [52] Germany	*N* = 13 (13 m) 22–31 y	1) Mg0 capsules 2) Mg0 effervescent tablets	Trt 1: 1) as single dose (450 mg Mg) Trt 2: 2) as single dose (450 mg Mg) Both cross‐over Trt 3: follow up for 4 weeks with the last taken Mg salt + usual diet (long‐term, not CO) **48 days** (1 trt day and 4 weeks trt)	Blood, Urine	24 h Urine: control, adaptation, follow‐up days On test days: Urine: 8–10 a.m., 10–12 a.m., 12 a.m.–2 p.m., 2–6 p.m., 6 p.m.–8 a.m. Blood: 8, 10, and 12 a.m., 2, 6, and 8 p.m.	AAS	Serum magnesium: unchanged Short‐Term fractional Mg excretion (mmol): 10 a.m. and later: sign. higher after 2) compared to 1) Cumulative 24 h‐excretion (mean and SE): 1) 5.21 ± 0.39, 2) 6.1 ± 0.56 (not sign.) Long‐term Urinary Mg Increase after Treatment 3: 1) sign. increase in the 1st week follow‐up 2) sign. increase all 4 weeks follow‐up BV (% ingested dose Mg, based on net excretion): 1) 4.7% vs. 2) 9.5%
Walker et al. (2003) [28] Great Britain	*N* = 51 (18 m, 33 w) 24.6–27.4 y	1) Mg‐amino acid chelate 2) MgCit 3) MgO 4) Placebo (cellulose) 5) Placebo (sorbitol)	Acute trt: 1)‐3): tabs. single dose (300 mg elemental Mg) 4), 5): 2 tabs. single dose Chronic 1)‐5): daily 1 month **60 days** (1 trt day, 1‐month trt)	Urine, Blood	Urine: 24 h (baseline, acute trt, end) Blood: fasting baseline, acute trt, end, each one in the morning	PM (Urine), AAS (blood)	Urinary Mg excretion: Acute and chronic: no sign. trt differences Chronic after adjustment of outliers: sign. higher increase after 1) and 2) compared to 3), 4), and 5) Mean and SEM Plasma Mg (mmol/L) 2) sign. increase to baseline 0.68 ± 0.03 after acute (0.73 ± 0.02) and chronic trt (0.72 ± 0.04), otherwise not sign. Erythrocyte Mg: no sign. difference between trt
Werner et al. (2019) [46] Slovakia	*N* = 14 (14 m) 23–56 y	1) elemental Mg 2) MgCit solution 3) Mg0 capsule	1): All participants 5 days before study 400 mg (to equalize Mg status) 2), 3): Single dose (400 mg elemental) single dose 1) or 2) **16 days** (1 trt day)	Blood, Urine	24 h urine: Tests days and the day after Fasting blood: day 0, 7 Blood: *t* = 2, 4, 8, 24 h	PM	Net 24 h Mg urinary excretion (mmol): Baseline: 5.33 ± 1.57 2) 6.6 ± 2.66 3) 6.02 ± 2.19, 2) higher than baseline, otherwise not sign. Plasma Mg increase (mmol/L): 2) sign. higher compared to 3) after 4 h (0.92 ± 0.06 vs. 0.88 ± 0.05) and 8 h (0.94 ± 0.06 vs. 0.90 ± 0.04) 2) led to significant increase at all times 3) never led to significant increase
White et al. (1990) [47] USA	*N* = 12 (9 m, 3 w) 23–46 y	1) MgCl syrup 2) Mg‐gluconate tab. 3) Slow‐Mg tables (MgCl and Ca‐diphosphate)	1) 16 mL MgCl in 30 mL syrup single dose 2) 14 tab. single dose 3) 6 tab. single dose Each 16 mmol Mg **26 days** (1 trt day)	Blood, Urine	All study days: Blood: *t* = 0, 1, 2, 3, 4, 8, 12, 24 Urine = pooled 0–4, 4–8, 8–12, 12–24 h	AAS	Total serum Mg (mmol/L): Overall, no sign. effect between 1)–3) over time Delta net AUC for Serum Mg: 1) 0.035 ± 0.112, 2) 0.012 ± 0.064, 3) –0.003 ± 0.047 mmol/L, 1) sign. higher 24‐h Mg excretion (mmol): 2) 0.66 ± 1.28, 3) 0.61 ± 1.07, sign. difference between 2) and baseline, otherwise not sign., no data for 1)
Wilimzig et al. (1996) [30] Germany	*N* = 16 m (16 m) 18–41 y	1) Mg_3_Cit_2_ granules 2) Mg_3_Cit_2_ granules 3) MgSo_4_ infusion 4) MgSo_4_ infusion	Group 1 (*n* = 8): 1) 12 mmol as single dose 2) 24 mmol as single dose Group 2 (*n* = 8): 3) 4 mmol, diluted in 0,9% NaCl 4) 8 mmol, diluted in 0,9% NaCl **24 days** (1 trt day)	Blood	Day before study: Over 12 h After administration: **Group 1**: *t* = 0 (shortly before), every half hour for the first 6 h, then every 2 h until t = 12 h **Group 2**: Every 1/4 h within the first 2 h, then every 1/2 h, then every 2 h until t = 12 h	AAS	Net Mg plasma concentration (compared to baseline): 1) increased 3.1% in 12 h 2) increased 4.6% in 12 h 3) increased 9.5% in 12 h 4) increased 16.1% in 12 h Oral: sign. increase in Mg plasma levels and AUC compared to baseline Parenteral: greater increase in Mg plasma levels and AUC

Abbreviations: AAS, atomic absorption spectrometry; BV, bioavailability; Cit, citrate; K, potassium; KCit, potassium citrate; KCl, potassium chloride; KMgCit, potassium magnesium citrate; M, men; Mg, magnesium; Mg0, magnesium oxide; MgCit, magnesium citrate; MgCl, magnesium chloride; MgOH_2_, magnesium hydroxide; NaCl, sodium chloride; ns, no specification; OES, optical emission spectroscopy; PM, photometric measurement; SD, standard derivation; SEM, standard error of mean; sign., significant; *t*, time; tab., tablets; trt, treatment; w, women.

^a^ Unless otherwise specified, the total administered dose (of all tab./capsules) is given in parentheses

^b^ Unless otherwise specified, trt days are the number of days for each trt

^c^ Unless otherwise specified, net excretion refers to the difference compared to the baseline values (e.g. control week, placebo)

^d^ Not corrected for baseline values

^e^ Healthy participants, the 4 participants with illnesses were excluded (The BV parameters were evaluated separately in the first part of the study, which was included in this review)

^f^ Self‐calculated (cumulative net excretion/administered dose * 100)

**TABLE 6:** Adverse effects observed in the studies on potassium salts.

Study	Adverse effects
Arnold et al. (1980) [42]	Diarrhea, stomach pain, abdominal cramps and nausea, vomiting 4 adverse effects from the tablet, 2 from the elixir and 1 from the capsule
Bechgaard and Shephard (1981) [38]	No data
Ben‐Ishay and Engelman (1973) [29]	Occult blood after KCl solution; Taste of the KCl solution: attributed as unbearable from all participants
Betlach et al. (1987) [39]	Minimal adverse effects according to the authors: one case of flatulence and two cases of mild stomachache
Caplain et al. (1991) [35]	Good tolerance, no side effects reported (according to abstract)
Dickerson and Bressler (1980) [33]	No data
Graffner and Sjögren (1971) [20]	No data
Harvey et al. (1989) [44]	No data
Holzgreve and Stephan (1978) [36]	Placebo: 1× nausea KCl retard tab. 1× nausea, 1× stomach pain and diarrhea, 1× poor compatibility KCl retard tab. enteric‐coated: 1× nausea, 1× stomach pain
Koenig et al. (1991) [18]	5 subjects: epigastric burning sensation, 1 subject: upper abdominal cramps after KCl
Li et al. (2023) [48]	60 adverse effects reported, 14 considered unrelated to the study drug Gastrointestinal diseases most common One case of creatine kinase increase
Lowance et al. (1982) [27]	KCl tab.: by physician 1× mild abdominal pain, by questionnaire: 3× abdominal pain, 4× diarrhea, 2× nausea KCl solution: by physician 4× abdominal pain, 1× nausea, 1× diarrhea; 2× abdominal pain, 2× heartburn, 2× diarrhea, 2× nausea, 1× headache; 11–12 bad taste
Melikian et al. (1988) [49]	Six men with abdominal pain, nausea, diarrhea, loose stools on the day of intervention
Mittapalli et al. (2017) [22]	No data
Möller et al. (1980) [26]	No data
Otto and Rooth (1973) [23]	No adverse effects
Rahkit et al. (1987) [41]	No data
Senel et al. (1991) [24]	No adverse effect
Skoutakis et al. (1979) [51]	Unpleasant taste for liquid K solution
Skoutakis et al. (1984) [34]	No data
Skoutakis et al. (1985) [50]	No adverse effects
Tannen and Cordano (1985) [31]	No adverse effects
Toner et al. (1985) [37]	Placebo and tablet: mild headache Syrup: unpalatable, caused nausea, epigastric burning, looseness of motion, soft stools (almost diarrhea)

**TABLE 7:** Adverse effects observed in the studies on magnesium salts.

Study	Adverse effects
Blancquaert et al. (2019) [19]	No data
Bøhmer et al. (1990) [53]	No adverse effects
Dualé et al. (2018) [21]	No side effect for either supplement
Firoz and Graber (2001) [43]	No gastrointestinal side effects
Gegenheimer et al. (1994) [40]	No data
Kappeler et al. (2017) [45]	No data
Koenig et al. (1991) [18]	No side effects
Lindberg et al. (1990a) [32]	No data
Lindberg et al. (1990b) [25]	No data
Siener et al. (2011) [52]	No data
Walker et al. (2003) [28]	No data
Werner et al. (2019) [46]	No data
White et al. (1990) [47]	No adverse effects
Wilimzig et al. (1996) [30]	Soft feces (12 mmol Mg: 3 volunteers 1/day; 24 mmol Mg: 2× once, 3× twice, 1× three times/day)

### 3.1 Dietary Requirements

All 23 studies on potassium salts gave dietary instructions with varying degrees of precision and strictness, while the amount and type of nutrients were inconsistent. Fifteen studies (65.2%) followed a standardized intake of potassium during the treatment days and/or prior to the study period (range: 18–100 mmol, median: 60 mmol, mean and SD: 61.9 ± 21.9 mmol) [22, 24, 26, 27, 29, 33, 35, 38, 39, 41, 42, 48, 49, 50, 51]. Nine studies specified the amount of energy/per day [27, 29, 35, 38, 39, 41, 42, 48, 51] while the energy intake (kcal/day) on treatment days between the studies varied between 1256 [27] and 4920 [42] kcal (median: 2300 kcal, mean and SD: 2508.4 ± 957.6). Twelve studies (52.2%), also specified the intake of other nutrients such as carbohydrates, proteins, fats, sodium, calcium, and chloride [18, 27, 33, 35, 39, 41, 42, 44, 48, 49, 50, 51]. Thirteen studies (56.5%) also defined the amount and type of liquid and/or drinks allowed [18, 22, 24, 27, 29, 31, 35, 37, 38, 41, 42, 44, 48].

All 14 studies on magnesium salts gave dietary instructions, although dietary magnesium intake was only standardized in five studies [25, 30, 32, 45, 52]. Nine studies also defined the liquid intake although the quantity was usually not specified [18, 19, 21, 25, 28, 32, 40, 45, 52]. In case information was given the magnesium content of the diet during the treatment days was between 200 and 747 mg (median: 247 mg).

### 3.2 Bioavailability

Twenty‐three studies examining potassium salts while the salt compound of interest for this review was KCl. One study additionally analyzed potassium citrate [18] while no studies on potassium carbonate and potassium sulfate were identified. Most studies (*n* = 20; 87%) compared the bioavailability of KCl from different administrations, for example, tablets with different release characteristics and solutions [20, 22, 23, 24, 27, 29, 31, 33, 34, 35, 36, 37, 38, 39, 41, 42, 44, 49, 50, 51]. The three other studies primarily compared salt compounds from different manufacturers [20, 26] (*n* = 2; 8.7%) or with potassium citrate as a salt compound [18] (*n* = 1; 4.4%). The potassium salt was usually administered as a single dose in *n* = 20 studies, all except for Graffner and Sjögren [20], Holzgreve and Stephan [36], and Otto and Rooth [23] which administered three [20, 36] or two times a day [23] over several days. Chronic dosing was investigated in a total of five studies, in some cases in addition to a single‐dose administration where the period of administration was 4 days [29], 5 days [20], or 7 days [31]. All studies examined the excretion of K^+^ in the urine, six studies (26.1%) additionally determined blood parameters [29, 31, 33, 34, 37, 41]. Flame photometry was used for K^+^ determination most frequently in eight studies (34.8%) [24, 27, 37, 38, 39, 42, 44, 49]. Other studies used flame spectroscopy (*n* = 1; 4.4%) [20], optical emission spectrometry (*n* = 2; 8.7%) [22, 48], photometric measurement [41] (*n* = 1; 4.4%), atomic absorption spectroscopy (*n* = 2; 8.7%) [23, 26], photometric measurement (*n* = 1; 4.4%), or Technicon Idee sample identification system (*n* = 1; 4.4%) [35]. No method was mentioned in the remaining six studies (26.1%) [18, 29, 31, 34, 36, 50]. The administered dose of K^+^ was between 20 and 80 mmol (mean: 49 mmol).

Most studies found no difference in KCl bioavailability across dosage forms. In 15 of 20 studies, cumulative urine excretion showed no significant difference on bioavailability between tablets, capsules, and solutions. One study focused on kinetics without comparing formulations. Four studies reported significant differences [22, 29, 42, 49]: Arnold et al. [42] found a higher excretion with KCl tablets than elixirs and capsules, Ben‐Ishay and Engelman [29] and Melikian et al. [49] observed higher bioavailability for solutions over tablets and capsules, while Mittapalli et al. [22] found that fast‐ and intermediate‐release tablets outperformed slow‐release tablets.

### 3.3 Quality Assessment

All 12 studies analyzing KCl kinetics found significant differences between formulations. Eight studies reported faster excretion for liquid forms (solution, syrup, elixir) over tablets or capsules [27, 29, 31, 33, 37, 39, 49, 50] while one study found the opposite [42], and another showed higher excretion for solid formulations at all time points [34]. In eight studies, kinetic comparisons were not possible due to missing or non‐extractable data [20, 22, 23, 34, 35, 36, 38, 41], though two qualitatively described an earlier excretion for liquids [34, 35]. No significant differences were found between KCl tablets from different manufacturers [40, 46] or between KCl, potassium citrate, and potassium magnesium citrate [18].

With regard to the relative values, defined as the total amount of potassium excreted as a proportion of the administered dose, the relative bioavailability of KCl capsules was 47.8% [49], 50.8% [42], 74.3% [50], or 79.0%[35]. The cumulative excretion in relation to the administered dose examined the bioavailability of the tablets ranged between 27.4% (KCl retard‐tablet single dose) [42] and 338.4% (KCl tablet single dose) [33] (median: 85%; mean ± SD: 85.2 ± 50.4). The bioavailability of a single dose KCl tablet has been calculated on the data provided in the text is substantially higher and implausible compared to other studies, where the next highest measured percentage is 102% (KCl retard tablet) [36]. This implausibly high bioavailability points toward methodological and reporting issues in the original study, which cannot be verified retrospectively. The bioavailability for liquid forms ranged from 52.7% (KCl elixier) [39] to 318.8% (KCl solution) [33] with a median of 69.5% and a mean of 100.8% ± 80.9% (SD). There was also an outlier of the maximum bioavailability of a KCl solution, which has also been self‐calculated on data provided in the text and looks implausible as the next highest value was 102.2% (KCl solution in black currant syrup) [20]. The total bioavailability of KCl from different preparations showed a mean bioavailability of 77.6% (median) and a range of 27.4%–338.8%. The average and standard deviation were 88.6% and 61.4%, respectively. One study examined the bioavailability of potassium citrate compared to KCl and potassium magnesium citrate, which showed a bioavailability of 52% for potassium citrate, which does not differ from the other potassium salts [18]. Two studies comparing different KCl formulations from different manufacturers found no significant differences [20, 48]. Blood parameters were measured in five studies, but two studies did not report outcomes [29, 41]. Three studies found a faster serum potassium increase with liquid versus solid KCl forms [31, 33, 34], while one reported a higher increase with KCl wax matrix tablets than the control [37].

The magnesium compounds investigated were magnesium oxide in nine studies [19, 25, 28, 32, 40, 43, 45, 46, 52], MgCl in four studies [21, 43, 47, 53], and magnesium citrate in five studies [25, 28, 32, 45, 46]. Magnesium sulfate [30] or carbonate [21, 40] were examined one or two times, respectively. The most common form of administration was a single dose in 11 studies while three studies [32, 43, 53] investigated split doses administered 2, 2–3, or 4 times per day. As part of a chronic testing, magnesium salts were administered over a period of 1 week [32] or 1 month [28, 52]. Some trials preceded magnesium supplementation to replenish magnesium stores [21, 30, 40, 45, 46], with one study [30] using a standardized magnesium‐rich diet instead of a supplement. Most studies examined urine (*n* = 13; 92.9%), except [30] and/or blood (*n* = 9; 64.3%) [19, 21, 25, 28, 30, 45, 46, 47, 52]. For the detection of magnesium, atomic absorption spectrometry was used in most studies (*n* = 10; 71.4%) [21, 25, 28, 30, 32, 40, 45, 47, 52, 53]. Other studies applied optical emission spectrometry (for urine), (*n* = 1; 7.1%) [45] or photometric measurement (*n* = 3; 21.4%) [19, 43, 46]. One study (7.1%) did not report any method [18]. The administered dose of magnesium ranged between 10.3 and 866 mg (median: 325 mg, mean ± SD: 360.7 ± 176.6 mg). In contrast to potassium salts, 13 out of the 14 studies (92.9%) compared different magnesium compounds with other magnesium salts which was not true for one study [52]. Due to the differences between various compounds in various combinations, the results of the studies on magnesium salts are difficult to compare. Magnesium oxide generally showed lower bioavailability than alternative magnesium salts. Seven of eight trials found it inferior [19, 25, 28, 40, 43, 45, 46], while one reported similar bioavailability to magnesium citrate [32]. One study showed higher bioavailability for effervescent capsules over tablets, with relative bioavailability of 4.7% and 9.5%, respectively [52]. Studies on MgCl show mixed results [21, 43, 47, 53], with relative bioavailability ranging from 9%–11% [43] to 30.1% (solution) [53] and 105.5% (tablet) [21]. Magnesium citrate results are difficult to compare due to varying study designs. Reported blood parameters also vary, including incremental AUC and maximum serum magnesium increase. With regard to these parameters five out of nine studies reported a lower bioavailability of magnesium oxide compounds compared to other compounds [19, 25, 28, 45, 46] while one study observed very high intra‐subject variability in serum and erythrocyte parameters [21]. Serum magnesium levels did not differ following the intake of magnesium capsules and effervescent tablets, respectively. MgCl compared to MgCl + calcium disphosphate and magnesium gluconate resulted in a higher delta of the AUC of the serum magnesium concentration [47]. A parenteral administration of magnesium sulfate caused a higher increase in magnesium plasma levels and AUC compared to an oral administration of magnesium citrate granules [30].

### 3.4 Adverse Effects

The adverse effects of potassium salts reported in the included studies are shown in Table 6. Fifteen out of 23 studies (65.2%) investigated adverse effects on potassium salts [18, 23, 24, 27, 29, 31, 35, 36, 37, 39, 42, 48, 49, 50, 51]. Side effects, including gastrointestinal symptoms and bad taste (especially of the liquid potassium salts), were reported in 10 studies (66.7%) [18, 27, 29, 36, 37, 39, 42, 48, 49, 51]. Other studies did not describe any side effects.

For magnesium salts, adverse effects reported in the included studies are listed in Table 7. Six out of 14 studies (42.9%) reported adverse effects on magnesium salts [18, 21, 30, 43, 47, 53]. Gastrointestinal symptoms as side effects were found in two studies (14.3%) [18, 30] while other studies did not report side effects.

All except two studies had a crossover design. These two were either completely in a parallel group design [28] or a sub‐study was in a parallel group design [31]. These two studies used a specified randomization method that can be considered appropriate [41, 48]. A carry‐over effect was evaluated in only five cases [24, 33, 40, 45, 51] and therefore, often classified as unclear. The risk of presenting the data only as a crossover sequence (item “unbiased data”) could either not be assessed due to a lack of data (was not apparent from the descriptions or figures) (*n* = 16) or was rated as low (*n* = 19). Only three studies [45, 50, 51] described the allocation concealment in detail, while 32 studies did not provide any information. Blinding was often unreported, leading to unclear bias in 22 cases. Seven studies [19, 21, 22, 36, 41, 45, 46] had a low and six [24, 27, 42, 43, 48, 50] a high risk for bias. Most studies (22 studies) had a low risk of incomplete outcome data, though 13 studies were unclear. Only a few cases reported selective outcomes, and therefore, the risk of bias was considered to be low in 30 studies. Other biases were unclear in 23 studies, with six cases each classified as high or low risk of bias. Justifications are listed in Table S4.

## 4 Discussion

This review evaluated the bioavailability and tolerability of magnesium and potassium salts as potential sodium chloride substitutes in human nutrition. The findings indicate that KCl exhibits adequate bioavailability, whereas magnesium oxide demonstrates comparatively lower bioavailability. Potassium citrate, magnesium citrate, and MgCl emerge as viable alternatives; however, gastrointestinal adverse effects and taste aversions, particularly with potassium salts, may present limitations.

### 4.1 Bioavailability of Potassium Salts

Most available data on KCl show sufficient bioavailability, ranging from 27.4% to 338.8% (median 77.6%), with no significant difference between KCl and potassium citrate [18]. Although the maximum value of 338.8% is implausible, the median value is less sensitive to outliers than the arithmetic mean and indicates that these two compounds have good bioavailability. Bioavailability appears unaffected by slow‐ or fast‐release forms [44], though liquid formulations generally show faster kinetics. Faster excretion may enhance short‐term bioavailability [42], but dietary intake with meals could alter absorption rates due to a slower release from a full stomach [11]. Variability among studies may stem from differences in study design, dietary intake, and participant potassium status. Inconsistent inclusion and exclusion criteria and unrecognized conditions could influence potassium balance. Dietary sodium, potassium intake, and insulin secretion may also affect excretion patterns, further complicating comparability [54]. Most formulations were tested on an empty stomach, whereas dietary potassium is typically consumed with meals, limiting applicability [55]. The methods of potassium salt administration varied, with some studies using single doses and others split doses over multiple days. Acute potassium intake leads to ∼50% urinary excretion while a large proportion is taken up intracellularly [56], which may be the reason for a higher bioavailability observed with chronic treatment with KCl retard tablets. Urine is, therefore, not the best matrix to measure potassium, as it may lead to an underestimation of true bioavailability. Circadian variations in electrolyte excretion have already been described in healthy men [57] may have led to further bias in potassium measurements. Frequent gastrointestinal side effects may impact adherence of the population to potassium salts. However, the high single doses of 20–80 mmol (782–3128 mg) used in bioavailability studies approach EFSA's daily recommended intake of 3.51 g [7]. This suggests that lower doses of dietary salt replacements may be better tolerated, which needs to be confirmed in further studies.

### 4.2 Bioavailability of Magnesium Salts

Magnesium oxide shows poor bioavailability, ranging from 4.7% to 9.5%, and is inferior to other magnesium salts. MgCl exhibits variable bioavailability (9.0%–105.5%), while magnesium citrate and carbonate show higher values (66.0% and 117.7%, respectively). Although magnesium sulfate has been investigated in a study [30], it has only been used as an intravenous reference salt, not allowing any statement on its bioavailability after oral intake. As a table salt alternative, MgCl and magnesium citrate are preferable to magnesium oxide due to better bioavailability. Organic magnesium compounds generally outperform inorganic ones. However, combining magnesium oxide with other salts, such as glycerophosphate [19], or using effervescent formulations may enhance its bioavailability [52]. The use of magnesium oxide as a table salt substitute may have a higher bioavailability compared to a tablet. Variability in study designs likely contributed to differences in magnesium bioavailability outcomes. Factors include participant numbers, magnesium concentrations, formulations, and the often‐unstated dietary magnesium intake. The magnesium loading phase, considered in only some studies, may have also affected the results. An unassessed baseline magnesium status could lead to a reduced excretion due to deficiency compensation. Since excretion reflects magnesium stores, lower excretion suggests depletion, while higher excretion indicates saturation [58]. Most of the studies included focused on men. However, one study included men and women and reported a higher baseline magnesium excretion in men, which suggests that sex differences significantly influence magnesium bioavailability and may account for the large variation in the overall study population [28]. Adverse effects in the studies were rare, suggesting a high tolerability of magnesium salts. However, daily doses ranged from 100.3 to 866.0 mg, sometimes exceeding the recommended intake of 300 mg for women and 350 mg for men [9].

The present review also has some limitations. The search criteria were slightly adjusted in 2024 due to limited author‐provided data on age and health status, leading to less strict inclusion criteria. Although it is not certain that all studies fully met the intended inclusion criteria, it is rather unlikely that pregnant women, for example, were included without being explicitly stated. This approach allowed for the inclusion of a higher number of studies. Despite all studies being randomized controlled trials having a similar study design, heterogeneity remains in some factors such as participant demographics, salt dosage, treatment duration, washout phases, and dietary restrictions. Sample sizes were relatively small (5–60 participants, mean: 14), although crossover designs require fewer participants than parallel group studies [59]. However, most studies lacked power analyses, and study quality was often suboptimal due to unclear bias risks from missing methodological details. The studies included only young, healthy adults, limiting the transferability to children, elderly persons, as well as pregnant or breastfeeding individuals. Age‐related gastrointestinal changes, reduced kidney function, and medication use can affect electrolyte balance and nutrient status [60], making the findings of the present review less applicable to the older population. The included studies span a long period of time, during which regulatory guidelines for RCT studies have evolved. Despite taste issues with some tested potassium formulations (especially solutions), potassium‐enriched dietary salts are generally better accepted [61]. Off‐tastes, such as bitterness, which may be relevant for consumer acceptance, can be reduced by mixing with NaCl [62], and potassium‐magnesium salt blends have also shown good acceptability [63]. This supports their bioavailability and practical applicability, which may give rise to a potential widespread implementation of salt alternatives in the food industry. However, it should be considered that not everyone can benefit from potassium substitutes. Potassium salts are unsuitable for individuals with kidney disease, potassium‐sparing medications, adrenal insufficiency, or uncontrolled diabetes, limiting their universal use [64]. This is particularly relevant for metabolic syndrome, where hypertension coexists with other cardiovascular risk factors [65]. Caution is also needed for patients with gastrointestinal conditions, as KCl supplements have been linked to mild to moderate gastrointestinal lesions [66]. Some of the studies included in this review observed gastrointestinal effects, while others showed no adverse effects. One potential explanation for this discrepancy is the varying methodologies employed in the administration of potassium salts across the included studies. According to the EFSA Panel, formulation appears to be a more relevant factor in gastrointestinal tolerance than dose [6]. A significant proportion of the included studies used potassium salts in tablet or liquid/solution form. Therefore, it is recommended that specific studies on the tolerability of salt forms used in the food industry are conducted in future. Of the studies included in the present review, only one reported mild gastrointestinal symptoms (soft feces) associated with magnesium salts. Nevertheless, it should be noted that the EFSA has set an UL of 250 mg per day for readily dissociable magnesium salts, as mild diarrhea has been observed in some individuals [9]. When used as a salt substitute, the dosage should be adjusted accordingly. The advantage of magnesium salts is that they appear to have no effect on potassium balance yet still demonstrate antihypertensive properties [10, 67]. Despite some limitations, bioavailable potassium and magnesium salts have the potential to be used in public health initiatives, given their antihypertensive and salt‐sparing effects. To ensure these benefits reach a large proportion of the population, information campaigns should address potential public concerns and give special consideration to vulnerable groups, such as those with chronic kidney disease [68].

## 5 Conclusion

Based on the present review, KCl can be suggested as a sodium chloride substitute with sufficient bioavailability. In this context, magnesium oxide seems to be less ideal compared to other magnesium salts. Potassium and magnesium citrate, as well as magnesium carbonate, show potential but require further high‐quality randomized controlled trials to confirm their bioavailability and their effects on blood pressure. Research on potassium carbonate, potassium sulfate, and magnesium sulfate is still lacking and needs to be addressed in future studies. Given the high prevalence of CVDs, reducing salt intake is crucial, and replacing sodium with potassium or magnesium salts could aid in blood pressure management and disease prevention.

## Conflicts of Interest

The authors declare no conflicts of interest.

## Supporting information



Supporting information

## Data Availability

Data sharing not applicable to this article as no datasets were generated or analyzed during the current study.
