# Supplementary material for: Bioavailability of Magnesium and Potassium Salts Used as Potential Substitutes for Sodium Chloride in Human Nutrition — A Review
Source: Mol Nutr Food Res. 2025 Sep 10;69(22):e70227. doi: 10.1002/mnfr.70227 (PMC12643194; doi:10.1002/mnfr.70227)
Supplement: Supplementary file 1 — Supporting information [file MNFR-69-e70227-s001.docx]

# Supplementary Material

1. **Summary of the refinement of the search strategy over the course of time**

**Supplementary Table 1 (STab.1)**. February and march 2023 – adjusted criteria (basis for CB's final search strategy)

| **Inclusion criteria** | **Exclusion criteria** |
| --- | --- |
| - Randomized controlled original studies - Healthy man and women from 18 to 65 - Selected magnesium or potassium salt compounds that could serve as salt substitute: Magnesium citrate, chloride, carbonate, sulfate, oxide and potassium citrate, chloride, carbonate - Human studies - Normokalemic and normomagnesic values - Oral administration of the salt substitutes as a supplement - Urine or blood parameters for evaluation | - Pregnancy or breastfeeding - Animal studies - Administration as drinks, individual foods |

**Supplementary Table 2 (STab.2).** August 2024 - specification of a formulation for explicitness (RM)

| **Inclusion criteria** | **Exclusion criteria** |
| --- | --- |
| - Randomized controlled original studies - Healthy men and women - Selected magnesium or potassium salt compounds that could serve as salt substitute: Magnesium citrate, chloride, carbonate, sulfate, oxide and potassium citrate, chloride, carbonate in comparison to an alternative salt substitute (may be other than those mentioned) or another formulation of the same salt compound e.g. fast vs. delayed or another manufacture - Human studies - Normokalemic and normomagnesic values or no indication of deviation - Oral administration of the salt substitutes as a supplement - Urine or blood parameters for evaluation | - Pregnancy or breastfeeding – inclusion if it was not explicitly described that pregnant/breastfeeding were included in the original study - Full text not available in German or English - Animal studies - Administration as drinks, individual foods |

1. **Quality assessment**


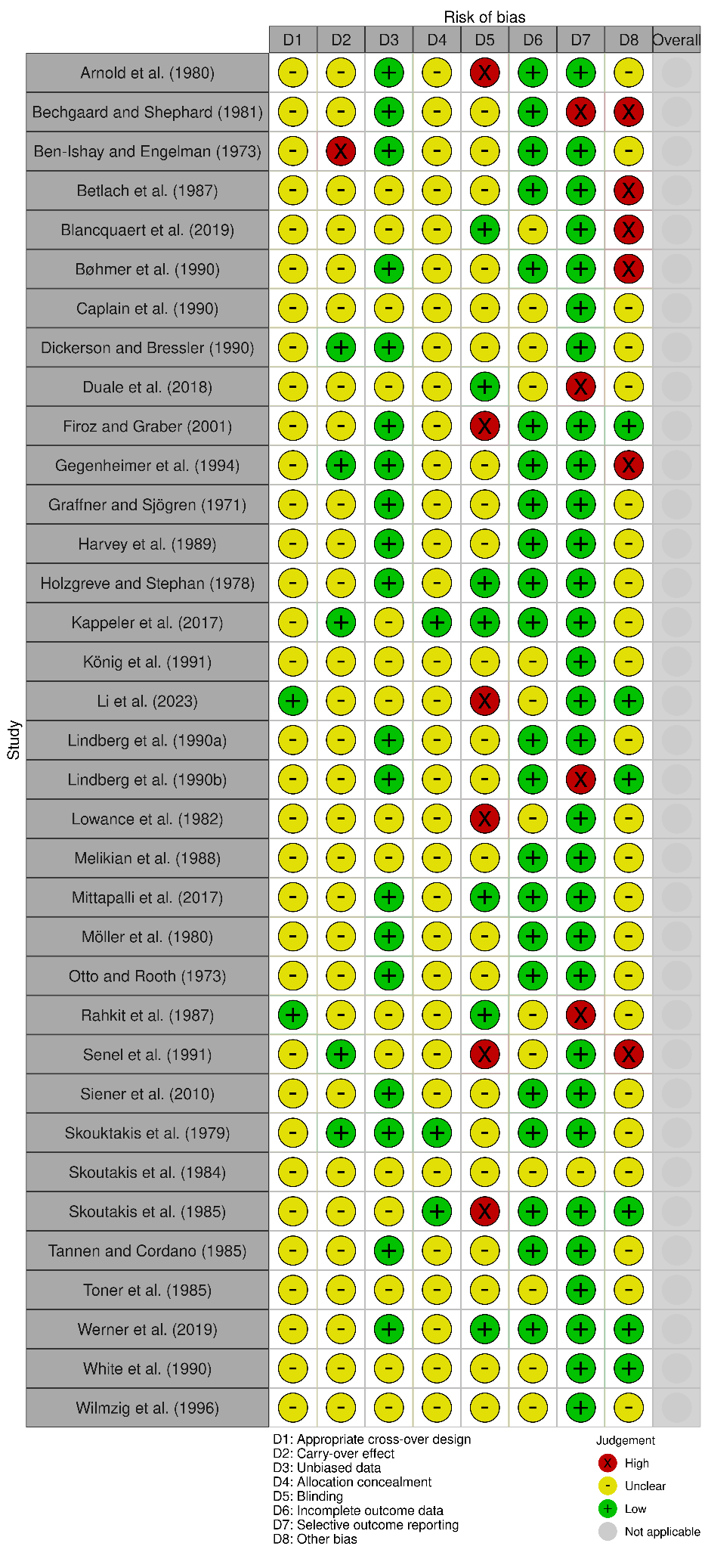


**Supplementary Figure 1 (SFig.1)*.*** Risk of bias of included Cross-over trials


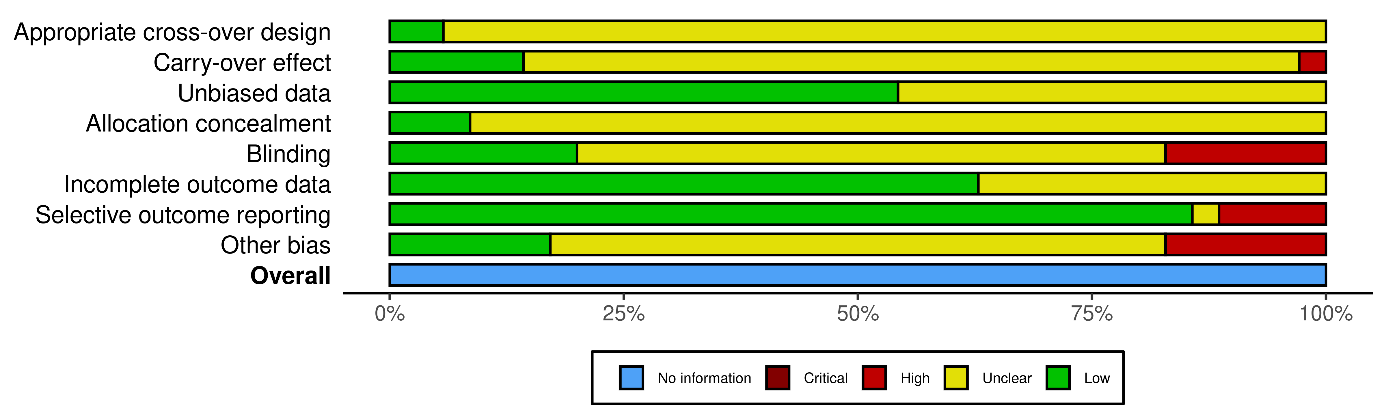


**Supplementary Figure 2 (SFig.2)***.* Summary Plot for Cross-over studies

**Supplementary Table 3 (STab.3)***.* Quality assessment for studies with parallel group design:

| **Study** | **A1** | **A2** | **A3** | **A4** | **A5** | **A6** | **A7** | **A8** |
| --- | --- | --- | --- | --- | --- | --- | --- | --- |
| Tannen et al. (1985) | Yes | Yes | Yes | Yes | No | Yes | NR | Yes |
| Walker et al. (2003)* | Yes | Yes | Yes | Yes | No | Yes | NR | Yes |

**Legend:**  A1: Cohort; A2: Control or comparison group; A3: Pre/ post intervention data; A4: Random assignment of participants to intervention; A5: Random selection of participants to intervention; A6: Random selection of participant assessment; A7: Follow up 80% or more; A8: Comparison groups equivalent at baseline on measures

* 1 part of the study was with Parallel group design

**Supplementary Table 4 (STab.4)***.* Justification assessment as items “Other bias” of the Cross-Over-Studies

| **Study** | **Other bias** | **Explanation** |
| --- | --- | --- |
| Arnold et al. (1980) | Unclear | Very high fluid intake, very high energy intake of 4920 kcal per day (not really convincing), overreporting of adverse effects possible, as subjects were told, to report any effect, also if it appears to be trivial |
| Bechgaard and Shephard (1981) | High | High intra individual variation in study 2, day to day variation in the diet in study 2 |
| Ben-Ishay and Engelman (1973) | Unclear | Different participant numbers for intervention, not for all data enough participants, group very selective (medical and veterinary students aged 23 to 25) |
| Betlach et al. (1987) | High | Problems with hourly urine specimens, interpretation of time intervals only possible to a limited extent |
| Blancquaert et al. (2019) | High | Only 6h measurement of Mg in serum, maybe excretion occurs also later during the timeframe |
| Bøhmer et al. (1990) | High | No limitation of physical activity, potential influence (unproven assumption) that healthy students in nutrition are in adequate Mg balance |
| Caplain et al. (1990) | Unclear | Only males |
| Dickerson and Bressler (1990) | Unclear | Only males |
| Duale et al. (2018) | Unclear | Only males |
| Firoz and Graber (2001) | Low | No further major limitations apparent |
| Gegenheimer et al. (1994) | High | Only males, no washout without Mg, diet not strictly controlled |
| Graffner and Sjögren (1971) | Unclear | Description of the study design very imprecise and difficult to understand, dietary regulations very vague, only males, only 9 participants |
| Harvey et al. (1989) | Unclear | 2,5 l distilled water on test days |
| Holzgreve and Stephan (1978) | Unclear | Only 6 participants, no information about sex |
| Kappeler et al. (2017) | Unclear | Only males |
| König et al. (1991) | Unclear | 3l distilled water on test days, electrolytic displacement possible |
| Li et al. (2023) | Low | No further major limitations apparent |
| Lindberg et al. (1990a) | Unclear | Only 7 participants for subanalysis, no power analysis, power is questionable |
| Lindberg et al. (1990b) | Low | No further major limitations apparent |
| Lowance et al. (1982) | Unclear | Only males |
| Melikian et al. (1988) | Unclear | Only males |
| Mittapalli et al. (2017) | Unclear | Only males |
| Möller et al. (1980) | Unclear | Diet seems to be very low caloric, might have influence on insulin and K |
| Otto and Rooth (1973) | Unclear | No statistic description |
| Rahkit et al. (1987) | Unclear | Only males, different data provided in abstract and text |
| Senel et al. (1991) | High | Control day without washout directly after testday |
| Siener et al. (2010) | Unclear | Only males |
| Skouktakis et al. (1979) | Unclear | Only males |
| Skoutakis et al. (1984) | Unclear | Very short description, no figures |
| Skoutakis et al. (1985) | Low | No further major limitations apparent |
| Tannen and Cordano (1985) | Unclear | Only males |
| Toner et al. (1985) | Unclear | Very small sample size |
| Werner et al. (2019) | Low | No further major limitations apparent |
| White et al. (1990) | Low | No further major limitations apparent |
| Wilmzig et al. (1996) | Unclear | Very small sample size |
